# Asociación entre la discapacidad física y la incidencia de síntomas depresivos en adultos mayores mexicanos

**DOI:** 10.7705/biomedica.5398

**Published:** 2020-12-09

**Authors:** Karen Luna-Orozco, Julián Alfredo Fernández-Niño, Claudia Iveth Astudillo-García

**Affiliations:** 1 Departamento de Salud Pública, Universidad del Norte, Barranquilla, Colombia Universidad del Norte Departamento de Salud Pública Universidad del Norte Barranquilla Colombia; 2 Servicios de Atención Psiquiátrica, Secretaría de Salud, Ciudad de México, México Escuela de Enfermería de la Secretaría de Salud del DF Secretaría de Salud México Mexico

**Keywords:** anciano, personas con discapacidad, depresión, evaluación de la discapacidad, incidencia, envejecimiento, estudios longitudinales, México, Aged, disabled persons, depression, disability evaluation, incidence, aging, longitudinal studies, México

## Abstract

**Introducción.:**

Las limitaciones funcionales asociadas con el proceso de envejecimiento pueden conducir al desarrollo de síntomas depresivos e incrementar la vulnerabilidad de los adultos mayores.

**Objetivo.:**

Estimar la asociación entre la discapacidad física y la incidencia de síntomas depresivos clínicamente significativos en adultos mayores mexicanos.

**Materiales y métodos.:**

Se hizo un estudio retrospectivo de cohorte con datos provenientes de la Encuesta Nacional sobre Salud y Envejecimiento en México (ENASEM). La muestra analítica (n=6.780) incluyó a adultos mayores de 50 años que contaran con mediciones de las variables principales y que no presentaran síntomas depresivos clínicamente significativos en la ronda cero. Estos síntomas se evaluaron con la escala CESD-9 y, la discapacidad, mediante el reporte de limitaciones para la realización de actividades básicas o instrumentales de la vida diaria. Se hicieron análisis descriptivos, bivariados y multivariados, utilizando el modelo de regresión logística y ajustando según las variables sociodemográficas, las condiciones de salud, las adversidades de la infancia, la participación social y los eventos vitales estresantes.

**Resultados.:**

La incidencia de síntomas depresivos clínicamente significativos fue de 25,75 % (IC_95_% 24,70-26,80). Comparados con aquellas personas sin limitaciones para las actividades instrumentales, se encontró un incremento del 68 % en el riesgo para el desarrollo de dichos síntomas (IC_95_% 1,10-2,57; p=0,015). En el modelo de actividades básicas de la vida diaria, la razón de probabilidad *(odds ratio,* OR) para su desarrollo fue de 1,36 (1,01-1,81; p=0,039), ambos ajustados por variables de confusión.

**Conclusión.:**

Las limitaciones en la vida diaria son un factor de riesgo importante para el desarrollo de síntomas depresivos clínicamente significativos en personas con seguimiento de dos años.

## Abstract (en)

**Introduction:**

Functional limitations associated with the aging process can lead to the development of depressive symptoms and increase the vulnerability of older adults.

**Objective:**

To estimate the association between physical disability and the incidence of clinically significant depressive symptoms in older Mexican adults.

**Materials and methods:**

We conducted a retrospective cohort study with data from the *Encuesta Nacional sobre Salud y Envejecimiento en México* (ENASEM). The analytical sample (n=6,780) included adults over 50 years old with measurements for the main variables and no clinically significant depressive symptoms reported in the first round. These symptoms were evaluated with the CESD-9 scale and disability by means of the report of activities of daily living (ADL) or instrumental activities of daily living (IADL). Descriptive, bivariate, and multivariate analyses were performed using logistic regression models adjusted by sociodemographic variables, health conditions, childhood adversities, social participation, and stressful life events.

**Results:**

The incidence of clinically significant depressive symptoms was 25.75% (95% CI: 24,70 - 26,80). Compared to those without IADL limitations, an increased risk of 68% for the development of clinically significant depressive symptoms was found (95% CI: 1.10-2.57; p= 0,015). With the ADL model, the OR for the development of clinically significant depressive symptoms was 1.36 (1.01 -1.81; p= 0.039). Both models were adjusted by confounding variables.

**Conclusion:**

Presenting limitations in daily life is an important risk factor for the development of clinically significant depressive symptoms at two years of follow-up.

El proceso de envejecimiento es una tendencia demográfica global; actualmente, la mayoría de las personas en buena parte del mundo puede aspirar a vivir más de 60 años ^1^. Sin embargo, como contrapeso de los beneficios del envejecimiento está el impacto de la evolución degenerativa del individuo en su entorno familiar y social y en los sistemas sanitarios, por lo que el fenómeno se ha convertido en un campo de interés para la salud pública. Entre los dominios más afectados se encuentran los relacionados con la pérdida de la funcionalidad y la independencia por discapacidad, así como las alteraciones en la esfera mental, específicamente los trastornos del espectro depresivo, que van desde la presencia de síntomas depresivos hasta episodios de franca depresión.

Si bien es cierto que el binomio discapacidad-depresión y su trayectoria han sido estudiados por diversos autores, es poca la información en el contexto latinoamericano. Además, aún existe controversia en cuanto a la direccionalidad de la asociación, toda vez que el comportamiento de estos fenómenos es diferente en los adultos mayores. En estos, aun cuando la depresión es menos frecuente, su evolución tiende a ser más tórpida, los síntomas tienden a hacerse crónicos y el número de recaídas es mayor en comparación con los adultos jóvenes ^2^.

El concepto de discapacidad se ha dinamizado con los años y hoy se la considera el resultado de la relación entre las condiciones del medioambiente y las alteraciones físicas, sensoriales o neurocognitivas, lo que en los adultos mayores se traduce generalmente en su dependencia funcional de otros ^3^^,^^4^. Sin embargo, bajo esta definición, un amplio espectro de condiciones podría considerarse como discapacidad, de allí las grandes diferencias en la manera de evaluar y clasificar dicho estado. En este contexto, tratando de superar esta barrera y dada la practicidad técnica para la medición, se ha extendido la valoración de la discapacidad en el adulto mayor con base en su independencia para la realización de las actividades básicas e instrumentales de la vida diaria.

Según el análisis de los datos obtenidos en la Encuesta sobre Salud, Bienestar y Envejecimiento en América Latina y el Caribe (SABE) del 2006, el porcentaje de adultos mayores con discapacidad varió considerablemente entre las regiones estudiadas, desde el 12 % en Montevideo hasta el 40,3 % en São Paulo, siendo la prevalencia promedio del 19 % ^5^. En el caso puntual de México, según *The study on global ageing and adult health* (SAGE) ^6^, la prevalencia de discapacidad entre el 2007 y el 2010 con base en la limitación para la realización de las actividades básicas de la vida diaria fue del 38,8 % entre adultos de 50 años o más. De hecho, se consideró que, por cada año más de vida, el 10 % de los adultos mayores que en el momento de la encuesta se encontraban libres de discapacidad requeriría ayuda para alguna de las actividades básicas de la vida diaria y se estimó que la incidencia podía ser mayor al evaluar la necesidad de asistencia para las actividades instrumentales ^7^. Como ya se mencionó, la discapacidad también está asociada con otras condiciones secundarias que afectan el estado de salud y la calidad de vida, como la depresión ^8^, que generalmente se subestima en el adulto mayor ^9^, por lo que el seguimiento de este grupo poblacional cobra importancia, ya que este factor incrementa su vulnerabilidad.

Históricamente se han descrito cuatro vías de aproximación a la relación entre las condiciones físicas de discapacidad y el desarrollo de la depresión. Desde la perspectiva biológica se plantea que, directa o indirectamente, ciertas afecciones físicas desencadenan cambios filológicos, especialmente neurohormonales, como sucede con la disminución de la concentración de monoaminas en los enfermos de Parkinson, ya que estas influyen en el desarrollo de la depresión por su efecto regulador del estado del ánimo. Por otro lado, las fluctuaciones anímicas se han relacionado indirectamente con los efectos adversos de medicamentos ^10^^,^^11^.

En cuanto a la perspectiva del comportamiento, esta considera las modificaciones de los comportamientos rutinarios de la vida diaria, por ejemplo, el abandono de hábitos, las restricciones laborales, las visitas frecuentes a centros asistenciales y el horario de la toma de medicamentos. Estos cambios en las conductas podrían llevar a percibir la enfermedad como un factor estresante, causa de angustia y depresión ^10^.

La perspectiva cognitiva plantea que la discapacidad se traduce en una situación coyuntural de choque emocional que hace necesaria una serie de ajustes, incluida la forma de relacionarse con el medio externo, de manera que aquellas personas con menor capacidad de adaptación y mayor tendencia a percibirse como inútiles y con poca esperanza en el futuro, tendrían mayor probabilidad de desarrollar depresión ^12^. La percepción de vulnerabilidad frente a la enfermedad contribuye a la construcción de patrones de pensamiento irracionales y da paso a una cascada de reacciones psicológicas negativas ^10^.

Por último, desde la perspectiva social se plantea que la discapacidad física y las enfermedades crónicas pueden deteriorar la relación entre el individuo y su medio externo, lo cual afecta sus redes de apoyo y hace cada vez más compleja la participación en actos sociales. Dado que las relaciones interpersonales son necesarias para la regulación de los estados afectivos y contribuyen a mantener formas positivas de pensamiento, la disrupción de las redes sociales aumenta el riesgo de depresión ^10^.

Partiendo de esta información, se reconocen diferentes orientaciones y tipos de asociaciones entre la discapacidad física y la depresión, y puede plantearse que la discapacidad funciona como un factor de estrés capaz de afectar el estado psicológico de los adultos mayores y desencadenar la depresión.

Hasta donde se sabe, no se ha estimado su efecto en estudios longitudinales en adultos mayores mexicanos, por lo que en este estudio se planteó estimar la asociación entre la discapacidad física y la incidencia a dos años de los síntomas depresivos clínicamente significativos en este grupo de población.

## Materiales y métodos

### Diseño de estudio

Se hizo un estudio retrospectivo de cohortes a partir de la ronda 0 y la ronda 1 de la Encuesta Nacional sobre Salud y Envejecimiento en México (ENASEM), como se explica a continuación.

### Población de estudio

La Encuesta Nacional sobre Salud y Envejecimiento en México (ENASEM) es un estudio prospectivo de salud representativo de 13 millones de mexicanos nacidos antes de 1951. La muestra se obtuvo en los 32 estados del país en zonas urbanas y rurales.

A la fecha se han realizado cuatro rondas: 2001, 2003, 2012 y 2015. Para la primera y segunda rondas, la muestra se selecciono a partir de los datos obtenidos mediante la Encuesta Nacional de Empleo correspondiente al cuarto trimestre del año 2000 ^13^. La ronda 0 incluyó una muestra de los mexicanos mayores de 50 años o más y sus parejas, independientemente de la edad de estas. En el 2003, se hizo un seguimiento en el cual se entrevistaron todos los participantes vivos y en condiciones de contestar las preguntas, un sustituto (familiar) de los participantes fallecidos, y un sustituto de aquellos que no pudieron completar su propia entrevista por enfermedad o ausencia temporal; además, se hizo una entrevista de base a la nueva pareja en caso de haberla. A pesar de contar con cuatro rondas y debido al número de pérdidas para el seguimiento entre el 2003 y el 2012, se utilizaron los datos de las rondas 0 y 1.

**Figura 1:**
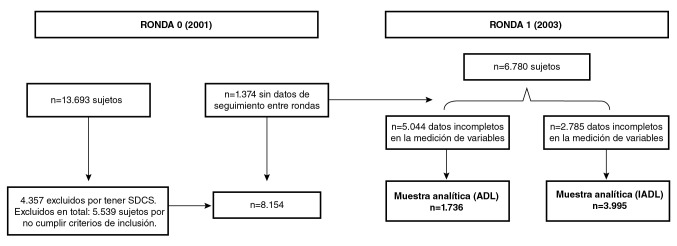
Diagrama de flujo de la conformación de la cohorte para las rondas 0 y 1 de la ENASEM

En el presente estudio se incluyeron aquellos sujetos de 50 años o más de la ronda cero que, además, contaran con mediciones de las variables principales en ambas rondas y hubieran contestado de forma directa y no mediante un sustituto. Se excluyeron los participantes con síntomas depresivos clínicamente significativos en la ronda 0. Al articular los datos de seguimiento entre las rondas 0 y 1 del grupo de 13.693 sujetos inicialmente encuestados, se encontró que 5.539 no cumplían con los criterios de inclusión. Entre los 8.154 restantes, hubo 1.374 pérdidas de seguimiento, quedando así una cohorte de 6.780 sujetos. Por último, la muestra analítica fue de 1.736 personas en el modelo de evaluación de la discapacidad mediante la valoración de la independencia para la realización de las actividades básicas de la vida diaria y de 3.995 personas en el modelo de medición de la discapacidad mediante el reporte de la independencia para la realización de las actividades instrumentales de la vida diaria. En la figura 1 se describe la conformación de la cohorte de estudio.

### Medidas

*Síntomas depresivos clínicamente significativos.* Se los definió como aquellos por encima de un umbral específico, aunque sin reunir los criterios necesarios para el diagnóstico de depresión mayor o de trastorno depresivo persistente ^14^; en este caso, la variable se evaluó en las dos rondas utilizando la escala *Center for Epidemiologic Studies Depression Scale* (CESD), modificada, de nueve ítems, que indaga sobre la presencia o ausencia de síntomas depresivos durante la semana anterior.

La escala se validó en el 2007 y se encontró una correlación estadísticamente significativa entre el cuestionario y el diagnóstico clínico de depresión. El punto de corte establecido en cinco o más tuvo una sensibilidad del 80,7 % y una especificidad del 68,7 % para el diagnóstico de depresión en el adulto mayor ^15^. Los individuos con síntomas depresivos clínicamente significativos en la ronda 0 fueron excluidos para garantizar la conformación de la cohorte con personas que no presentaban el resultado sobre el que se inquiría.

*Discapacidad.* La discapacidad física se evaluó mediante el reporte de limitaciones para la realización de las actividades básicas e instrumentales de la vida diaria. Las escalas utilizadas fueron la de Katz y la escala de Lawton y Brody ^16^. Con la escala de Katz (para las actividades básicas) se valoraron las limitaciones para bañarse, ir al baño, trasladarse fuera de la cama o silla sin ayuda, vestirse y comer, así como el control de esfínteres. Por otro lado, con la de Lawton y Brody (para las actividades instrumentales) se analizó la necesidad de asistencia para preparar alimentos, tomar medicamentos, ir de compras y administrar el dinero. La presencia de discapacidad se planteó en los casos en los que se reportó limitación para la ejecución, por lo menos, de una de estas actividades.

*Covariables.* Los modelos se ajustaron según las variables relacionadas con la presencia de discapacidad y de síntomas depresivos clínicamente significativos, incluidos los datos sociodemográficos, el estado de salud, las redes de apoyo, las adversidades de la infancia y los eventos vitales estresantes.

Las variables sociodemográficas incluyeron el estado civil (soltero, casado, viudo, unión libre, separado), el grupo de edad (50-59, 60-69, 70-79, 80 o más años), la escolaridad (ninguna, primaria, secundaria, estudios superiores), el sexo y el propio reporte sobre la situación económica (muy buena, buena, regular, mala). La presencia de adversidades en la infancia se evaluó con base en la valoración de las instalaciones sanitarias en el interior del hogar antes de los 10 años, concebida esta como símil de la pobreza infantil según se plantea en los documentos de la UNICEF en los que se la ha utilizado para analizar la dimensión socioeconómica, conjuntamente con otras como nutrición, salud y educación ^17^.

En la valoración de las variables relacionadas con el estado se salud, se utilizó el propio reporte de los entrevistados sobre diabetes mellitus, obesidad, hipertensión arterial sistémica, enfermedad pulmonar (asma o EPOC), cáncer, cardiopatía isquémica, artritis, enfermedad cerebrovascular, déficit cognitivo, percepción del propio estado de salud y el antecedente de fracturas.

La obesidad se determinó según el índice de masa corporal (IMC) con base en el peso y la talla reportadas por los propios participantes y se clasificó como normal (IMC: 18,5 a 24,9 kg/m^2^), sobrepeso (IMC: 25 a 29,9 kg/m^2^) u obesidad (IMC: ≥30 kg/m^2^). Para la categorización del deterioro cognitivo, se utilizó la versión reducida del *Cross-Cultural Cognitive Examination,* cuyos resultados se dividieron con base en un punto de corte de 39 o menos (presencia de deterioro) y 40 o más (ausencia de deterioro) ^18^. En cuanto a la presencia de "multimorbilidad" se consideró que esta existía cuando la persona reportaba padecer dos o más enfermedades crónicas ^19^.

La participación social se midió con base en el reporte de la participación en actividades de voluntariado, considerando que la intervención en eventos que promueven la integración social puede contribuir a mejorar la salud mental de este grupo de personas al brindarles oportunidades para mantener y desarrollar relaciones interpersonales que les permiten ser parte activa de su núcleo social y familiar ^20^^,^^21^.

Por último, la evaluación de los eventos vitales estresantes se basó en tres de los aspectos de la escala de reajuste social adaptada para adultos mayores de Holmes y Rahe ^22^: separación marital, muerte del cónyuge y muerte de un familiar cercano, en este caso, los hijos.

### Análisis estadístico

Inicialmente, se hizo un análisis descriptivo de todas las variables utilizando la mediana como medida de tendencia central y el rango intercuartil como medida de dispersión para las variables cuantitativas, y las proporciones y un intervalo de confianza (IC) del 95 % para las variables cualitativas.

Para el análisis bivariado de las asociaciones entre la variable dependiente y cada una de las variables independientes, se estimó la razón de momios *(odds* ratio, OR) con su respectivo IC de 95 % y el valor de p. En cuanto al análisis multivariado, se usaron modelos de regresión logística. Los resultados se presentan como OR, con sus respectivos IC del 95 % y el valor de p.

En el modelo 1 se evaluó la asociación entre la discapacidad para realizar las actividades instrumentales de la vida diaria como variable explicativa principal y la incidencia de los síntomas depresivos clínicamente significativos como variable dependiente, ajustada por las variables sociodemográficas, la historia de eventos vitales estresantes, las adversidades en la infancia, la participación social y las variables relacionadas con el estado se salud: hipertensión arterial sistémica, diabetes mellitus, asma o EPOC, enfermedad cerebrovascular, infarto agudo de miocardio, artritis, fractura, obesidad, cáncer, "multimorbilidad" percepción del propio estado de salud y déficit cognitive.

**Figura 2:**
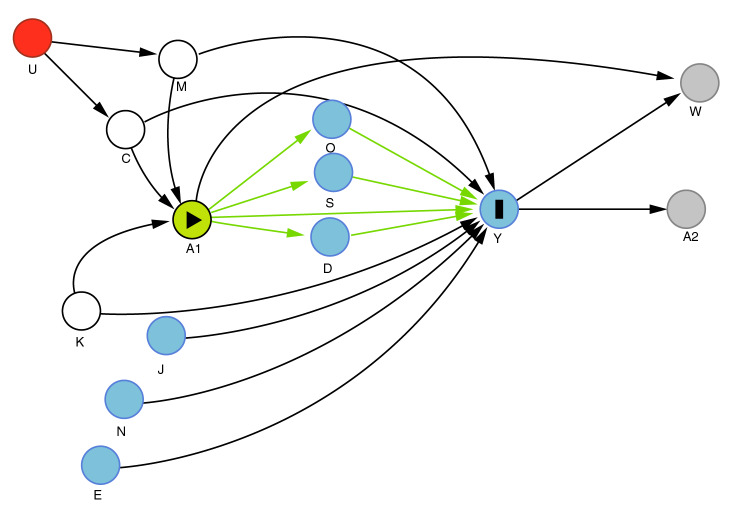
Diagrama aciclico dirigido utilizado para establecer potenciales variables de confusion de la relacion entre discapacidad y Depresión A1: Discapacidad 1; Y: Sintomas depresivos; A2*: Discapacidad 2 M: multimorbilidad (como constructo); C: morbilidad asociada (ver la lista completa en la seccion de materiales y metodos); N: nivel de ingresos; E: eventos vitales; W: perdidas en el seguimiento; K: adversidades en la infancia; S: percepcion de la propia salud; D: dolor (sin datos de medicion); O: aislamiento social (sin datos de medicion); J: red deficiente de apoyo familiar (sin datos de medicion); U: predisposicion genetica, estilo de vida (sin datos de medicion)

En el modelo 2 se utilizó la presencia de limitaciones para la realización de actividades básicas de la vida diaria como variable independiente principal ajustada por las mismas variables del modelo 1. La inclusión de las covariables descritas y la especificación de los modelos se ajustaron a las relaciones reportadas en la literatura especializada, y el potencial papel de las principales variables de confusión de la relación, mediante la construcción de un diagrama acíclico dirigido utilizando el paquete DAGitty (figura 2). Todos los análisis estadísticos se hicieron con el paquete SPSS™, versión 22.0 (figura 2).

## Resultados

### Análisis exploratorio

Con respecto a las características básales de la cohorte, la mediana para la edad fue de 59 años (Q_1_-Q_3_: 54-67), el 74,1 % estaba casado o vivía en unión libre; el 20,7 % no tenía ningún grado de escolaridad; el 48,68 % correspondía a mujeres y el 64,7 % manifestó una situación económica regular.

En cuanto al estado de salud, el 15,3 % del total de sujetos que contaba con la medición de las limitaciones para la realización de las actividades básicas de la vida diaria se consideró con discapacidad y, al evaluar la misma variable con base en las actividades instrumentales, el 3,4 %.

En lo que respecta a las enfermedades cardiometabólicas consideradas en el estudio, el 33,2 % reportó antecedentes de hipertensión arterial sistémica; el 13,5 % manifestó ser diabético; el 24,2 % tenía obesidad según su IMC; el 2,5 % había sufrido un infarto agudo de miocardio, y el 2,0 %, una enfermedad cerebrovascular. Además, el 5,0 % tenía morbilidades respiratorias como EPOC o asma; el 1,6 %, cáncer; el 16,6 %, artritis; el 11,4 % se había fracturado, y el 51,8 % tenía déficit cognitivo.

En relación con los eventos vitales estresantes, el 14,5 % había sufrido la pérdida de su cónyuge; el 7,4 % estaba separado, y el 35,5 % había perdido a uno de sus hijos.

Por otra parte, el 64,5 % de la cohorte había tenido adversidades en su infancia y el 14,4 % participaba activamente en actividades de voluntariado.

En el cuadro 1 se detallan las características sociodemográficas, así como las relacionadas con el estado de salud y otras covariables discriminadas por sexo. En cuanto a la incidencia de síntomas depresivos clínicamente significativos, a los dos años de seguimiento esta fue del 25,75 % (IC_95_% 24,70-26,80).

**Cuadro 1:** Características basales de la cohorte de mexicanos de 50 años o más (ENASEM, ronda 0*)

Variables		Mujeres n=3.301 (%)	Hombres n=3.479 (%)
Sociodemográficas			
Edad (años)	50-59	1.707 (51,7)	1.725 (49,6)
	60-69	1.007 (30,5)	1.076 (30,9)
	70-79	461 (14,0)	548 (15,8)
	≥80	126 (3,8)	130 (3,7)
Estado civil	Soltero	180 (5,5)	85 (2,4)
	Casado	1.904 (57,7)	2.729 (78,4)
	Unión libre	126 (3,8)	268(7,7)
	Separado	338 (10,2)	166 (4,8)
	Viudo	753 (22,8)	231 (6,6)
Escolaridad	Ninguna	742 (22,5)	663 (19,1)
	Primaria	1.757 (53,2)	1.926 (55,4)
	Secundaria	198 (6,0)	267 (7,7)
	Estudios superiores	604 (18,3)	619 (17,8)
Reporte de los propios participantes sobre su situación Buena económica	Muy buena	76 (2,4)	82 (2,3)
	Buena	786 (23,9)	695 (20,1)
	Regular	2.112 (64,2)	2.258 (65,2)
	Mala	316 (9,6)	427 (12,3)
Relacionadas con el estado de salud			
Actividades instrumentales de	Sin discapacidad	3.083 (95,4)	2.970 (97,8)
la vida diaria	Con discapacidad	149 (4,6)	67 (2,3)
Actividades básicas de la vida	Sin discapacidad	1.401 (86,2)	1.002 (82,7)
diaria	Con discapacidad	224 (13,8)	210 (17,3)
Multimorbilidad	Sí	1.098 (53,6)	1.054 (41,6)
Hipertensión	Sí	1.289 (39,9)	891 (26,6)
Diabetes mellitus^**^	Sí	477 (14,8)	412 (12,3)
Cáncer^**^	Sí	78 (2,4)	26 (0,8)
EPOC y asma^**^	Sí	164 (5,1)	163 (4,9)
Infarto agudo de miocardio^**^	Sí	58 (1,8)	106 (3,2)
Enfermedad cerebrovascular^**^	Sí	59 (1,8)	72 (2,2)
Artritis^**^	Sí	657 (20,4)	435 (13,0)
Fractura^**^	Sí	394 (12,2)	364 (10,7)
Obesidad	IMC≥30 kg/m^2^	673 (28,9)	609 (20,5)
Déficit cognitivo	Positivo<40 puntos	1.591 (53,9)	1.534 (49,8)
Percepción de la propia salud	Excelente	72 (2,2)	105 (3,0)
	Muy buena	144 (4,4)	241 (6,9)
	Buena	1.217 (36,9)	1.419 (40,8)
	Regular	1.597 (48,4)	1.434 (41,2)
	Mala	271 (8,2)	279 (8,0)
Eventos vitales estresantes	Fallecimiento de un hijo	1.164 (37,5)	1.114 (33,7)
	Separación marital	338 (10,2)	166 (4,8)
	Viudez	753 (22,8)	231 (6,6)
Adversidades de la infancia	Sí	2.007 (61,2)	2.334 (67,6)
Participación social	Participación en actividades de voluntariado (-)	2.777 (84,2)	3.009 (87,0)

* Se discriminó el porcentaje de cada variable con respecto al número efectivo de participantes por variable. Se presentan las características basales (ronda 0) de la muestra analítica de estudio descrita en materiales y métodos.

** Según informe de los propios participantes

En el análisis bivariado se encontró una asociación significativa entre la presencia de discapacidad, medida mediante la evaluación de las limitaciones para las actividades básicas de la vida diaria (p<0,001) y las actividades instrumentales (p<0,001), y la presencia de "multimorbilidad" (p<0,001), hipertensión arterial sistémica (p<0,001), artritis (p<0,001), enfermedad cerebrovascular (p<0,001), déficit cognitivo (p<0,001), EPOC o asma (p=0,017) y antecedentes de fractura (p=0,013).

Asimismo, se demostró una asociación significativa y un incremento de la fuerza de asociación cuando la percepción de la propia situación económica y el estado de salud era desfavorable. El sexo femenino, la edad mayor o igual a 60 años, no haber tenido educación o solo la primaria, las adversidades en la infancia, la pérdida del cónyuge y la no participación en actividades de voluntariado, también se asociaron significativamente con la variable principal (cuadro 2).

**Cuadro 2:** Análisis bivariado de la incidencia de los síntomas depresivos clínicamente significativos y las características clínicas y sociodemográficas de la muestra (ENASEM, rondas 0 y 1)

Variables		Presencia de SDCS n=1.746 (%)	OR (IC_95%_) ^*^	p
Sociodemográficas				
Edad (años)	50-59	796 (23,2)	Referencia	
	60-69	554 (26,6)	1,2 (1,0-1,3)	0,004
	70-79	306 (30,3)	1,4 (1,2-1,6)	<0,001
	≥80	90 (35,2)	1,7 (1,3-2,3)	<0,001
Sexo	Hombre	724 (20,8)	Referencia	<0,001
	Mujer	1.022 (31,0)	1,70 (1,52-1,90)	
Estado civil	Soltero	68 (25,7)	1,0 (0-8-1,4)	0,589
	Casado	1.121 (24,2)	Referencia	
	Unión libre	106 (26,9)	1,1 (0,9-1,4)	0,230
Escolaridad	Ninguna	478 (34,0)	3,0 (2,4-3,6)	<0,001
	Primaria	1.013 (27,5)	2,2 (1,8-2,6)	<0,001
	Secundaria	75 (16,1)	1,1 (0,8-1,5)	0,418
	Estudios superiores	178 (14,6)	Referencia	
Situación económica^**^	Muy buena	16 (10,1)	Referencia	
	Buena	276 (18,6)	2,0 (1,1-3,4)	0,009
	Regular	1.181 (27,0)	3,2 (1,9-5,5)	<0,001
	Mala	263 (35,4)	4,8 (2,8-8,3)	<0,001
Estado de salud				
Actividades instrumentales de la vida diaria	Sin discapacidad	1.514 (21,0)	Referencia	
	Con discapacidad	95 (32,4)	2,3 (1,7-3,0)	<0,001
Actividades básicas de la vida diaria Sin discapacidad	Sin discapacidad	726 (25,3)	Referencia	
	Con discapacidad	172 (31,5)	1,5 (1,2-1,8)	<0,001
Presencia de multimorbilidad	No	438 (15,0)	Referencia	<0,001
	Sí	596 (23,6)	1,7 (1,5-2,0)	
Hipertensión arterial^***^	No	1.032 (19,5)	Referencia	<0,001
	Sí	664 (25,6)	1,4 (1,2-1,5)	
Cáncer^***^	No	1.676 (21,6)	Referencia	0,671
	Sí	25 (20,5)	0,9 (0,5-1,4)	
Diabetes mellitus^***^	No	1.445 (21,3)	Referencia	0,056
	Sí	253 (23,2)	1,1 (0,9-1,3)	
EPOC y asma^***^	No	1.597 (21,3)	Referencia	0,017
	Sí	103 (26,5)	1,3 (1,0-1,7)	
Artritis^***^	No	1.307 (19,8)	Referencia	0,013
	Sí	391 (30,5)	1,7 (1,5-2,0)	
Fractura^***^	No	1.474 (20,9)	Referencia	0,013
	Sí	222 (24,6)	1,2 (1,0-1,4)	
Enfermedad cerebrovascular^***^	No	1.643 (21,3)	Referencia	<0,001
	Sí	54 (34,6)	2,0 (1,4-2,9)	
Infarto agudo de miocardio^***^	No	1.654 (21,5)	Referencia	0,512
	Sí	46 (22,3)	1,1 (0,7-1,5)	
Obesidad	No	928 (19,1)	Referencia	0,136
	Sí	322 (21,3)	1,1 (0,9-1,2)	
Déficit cognitivo	No	593 (17,1)	Referencia	
	Sí	906 (24,6)	1,5 (1,4-1,7)	<0,001
Percepción de la propia salud	Excelente	14 (6,7)	Referencia	
	Muy buena	56 (11,7)	1,9 (1,0-3,6)	0,029
	Buena	518 (16,2)	2,8 (1,6-4,9)	<0,001
	Regular	924 (22,2)	5,1 (2,9-8,8)	<0,001
	Mala	234 (34,9)	8,6 (4,8-15,2)	<0,001
Adversidades de la infancia	No	472 (19,8)	Referencia	
	Sí	1.260 (29,0)	1,6 (1,4-1,8)	<0,001
Eventos vitales estresantes				
Separación marital	No	1.602 (25,5)	Referencia	0,133
	Sí	144 (28,6)	1,1 (0,9-1,4)	
Viudez	No	1.439 (24,8)	Referencia	
	Sí	307 (31,2)	1,3 (1,1-1,5)	<0,001
Fallecimiento de un hijo	No	955 (23,1)	Referencia	
	Sí	692 (30,4)	1,4 (1,2-1,6)	<0,001
Participación social	No	1.523 (26,3)	1,2 (1,0-1,4)	0,007
	Sí	216 (22,2)	Referencia	

SDCS: síntomas depresivos clínicamente significativos

* Se discriminó el porcentaje de cada variable con respecto al número efectivo de participantes por variable.

^**^ Estimador crudo

^***^ Según informe de los propios participantes

### Análisis multivariado

En el modelo 1, la incidencia de síntomas depresivos clínicamente significativos y la discapacidad se asociaron con un incremento de la probabilidad para el desarrollo de este evento del 68 % (OR=1,68; IC_95%_ 1,10-2,57; p=0,015) comparado con los sujetos sin discapacidad. También, hubo asociación significativa con el sexo (OR=1,77; IC_95%_ 1,50-2,11; p<0,001), una edad superior a los 80 años (OR=1,91; IC_95%_ 1,15-3,19; p=0,012), una buena percepción de la propia salud (OR=2,02; IC_95%_ 1,03-3,94; p=0,039), una percepción regular (OR=2,95; IC_95_% 1,50-5,76; p=0,002) y una mala percepción (OR=5,18; IC_95%_ 2,54-10,56; p<0,001), así como con la ausencia de escolaridad (OR=1,47; IC_95%_ 1,07-2,01; p=0,015) y con tener solo primaria (OR=1,29; IC_95%_ 1,01-1,63; p=0,036), siendo el comparador en esta covariable la categoría de estudios superiores.

En el modelo 2, a pesar de la reducción importante del tamaño de la muestra (n=1.736), se encontró coherencia en la asociación significativa entre la discapacidad y la incidencia de síntomas depresivos clínicamente significativos (OR=1,36; IC_95%_ 1,01-1,81; p=0,039). Además, se mantuvo la asociación con covariables como el sexo (OR=1,51; IC_95%_ 1,18-1,93; p<0,001), la ausencia de escolaridad (OR=1,91; IC_95%_ 1,23-2,97; p<0,004), la percepción regular (OR=9,25; IC_95%_ 1,23-69,15; p=0,030) y una mala percepción (OR=15,45; IC_95%_ 2,03-117,54; p=0,008) del propio estado de salud, sin embargo, en estos estratos de la variable la estimación de la relación fue sustancialmente imprecisa. En el cuadro 3 se presentan estos resultados con mayor detalle.

**Cuadro 3:** Regresión logística binaria para los síntomas depresivos clínicamente significativos en mexicanos de 50 años o más (ENASEM, rondas 0 y 1)

Variables		Modelo 1 (n=3.995) OR (IC_95%_)	p	Modelo 2 (n=1.736) OR (IC_95%_)	p
Discapacidad		^*^1,68 (1,10-2,57)	0,015	^*^1,36 (1,01 -1,81)	0,039
Estado civil	Casado	Referencia		Referencia	
	Soltero	1,27 (0,68-2,36)	0,445	1,32 (0,53- 3,27)	0,550
	Unión libre	1,19 (0,83-1,69)	0,333	1,08 (0,65-1,80)	0,754
Edad (años)	50-59	Referencia		Referencia	
	60-69	1,00 (0,84-1,21)	0,927	0,97 (0,75-1,25)	0,817
	70-79	1,12 (0,87-1,44)	0,372	0,88 (0,62-1,24)	0,468
	80 o más	^*^1,91 (1,15-3,19)	0,012	1,67 (0,91-3,07)	0,096
Escolaridad	Ninguna	^*^1,47 (1,07-2,01)	0,015	^*^1,91 (1,23-2,97)	0,004
	Primaria	^*^1,29 (1,01-1,63)	0,036	1,34 (0,95-1,90)	0,088
	Secundaria	0,75 (0,52-1,08)	0,132	0,87 (0,51-1,50)	0,637
	Superior	Referencia		Referencia	
Sexo femenino		^*^1,77 (1,50-2,11)	<0,001	^*^1,51 (1,18-1,93)	0,001
Multimorbilidad		1,09 (0,83-1,43)	0,508	1,14 (0,79-1,65)	0,475
Hipertensión		1,13 (0,93-1,38)	0,197	1,05 (0,81-1,37)	0,699
Cáncer		0,59 (0,30-1,14)	0,120	0,49 (0,20-1,18)	0,114
Diabetes mellitus		0,86 (0,68-1,09)	0,233	0,80 (0,59-1,09)	0,165
EPOC y asma		0,98 (0,70-1,38)	0,932	1,11 (0,74-1,66)	0,600
Artritis		1,21 (0,97-1,51)	0,085	1,11 (0,85-1,45)	0,432
Fractura		0,86 (0,66-1,12)	0,275	0,90 (0,65-1,24)	0,547
Enfermedad cerebrovascular		1,48 (0,87-2,52)	0,147	1,56 (0,86-2,83)	0,140
Infarto agudo de miocardio		0,88 (0,54-1,43)	0,621	0,67 (0,38-1,18)	0,169
Obesidad		0,99 (0,81-1,21)	0,995	0,92 (0,70-1,20)	0,533
Déficit cognitivo		1,04 (0,85-1,26)	0,672	0,95 (0,72-1,25)	0,749
Percepción de la propia salud	Excelente	Referencia		Referencia	
	Muy buena	1,68 (0,78-3,59)	0,178	3,13 (0,36-27,22)	0,299
	Buena	^*^2,02 (1,03-3,94)	0,039	6,74 (0,89-50,63)	0,064
	Regular	^*^2,95 (1,50-5,76)	0,002	^*^9,25 (1,23-69,15)	0,030
	Mala	^*^5,18 (2,54-10,56)	<0,001	^*^15,45 (2,03-117,54)	0,008
Eventos vitales estresantes	Separación marital	1,30 (0,99-1,71)	0,053	1,27 (0,85-1,91)	0,232
	Viudez	1,16 (0,92-1,47)	0,206	1,20 (0,87-1,65)	0,258
	Fallecimiento de un hijo	1,06 (0,89-1,26)	0,471	1,07 (0,84 -1,36)	0,548
Adversidades de la infancia		1,17 (0,98-1,40)	0,077	1,07 (0,83-1,38)	0,572
Participación social		1,16 (0,93-1,45)	0,169	1,25 (0,92-1,68)	0,147
Percepción de la propia situación económica	Muy buena	Referencia		Referencia	
	Buena	1,08 (0,58-2,02)	0,797	1,53 (0,55-4,21)	0,409
	Regular	1,40 (0,76-2,59)	0,279	1,52 (0,56-4,12)	0,407
	Mala	1,70 (0,88-3,25)	0,110	1,76 (0,62-4,97)	0,283

Referencias para las categorías dicotómicas: sexo masculino; no tener discapacidad, hipertensión arterial sistémica, diabetes mellitus, enfermedad cerebrovascular, infarto agudo de miocardio, artritis, historia de fracturas, déficit cognitivo, enfermedades respiratorias (EPOC o asma) u obesidad; no ser separado ni viudo; no haber padecido el fallecimiento de un hijo ni adversidades en la infancia y no participar en actividades de voluntariado. * p<0,05 Modelo 1, variable explicativa principal: limitaciones para la realización de actividades instrumentales de la vida diaria Modelo 2, variable explicativa principal: limitaciones para la realización de las actividades básicas de la vida diaria

## Discusión

Los resultados del presente estudio sustentan la asociación entre la presencia de discapacidad física y el desarrollo de síntomas depresivos clínicamente significativos, aunque ajustados por potenciales factores de confusión como la coexistencia de otras morbilidades, la percepción del propio estado de salud, el padecimiento de adversidades en la infancia y otras variables como las sociodemográficas, las relacionadas con la participación social y la historia de eventos vitales estresantes.

Cabe destacar el hallazgo de una incidencia de síntomas depresivos clínicamente significativos del 25,75 %, así como la asociación significativa entre el sexo femenino, la percepción del propio estado de salud y la ausencia de escolaridad con el desarrollo de síntomas depresivos clínicamente significativos en los dos modelos multivariados. En la práctica, se considera que el efecto de la discapacidad sobre la incidencia de la depresión se debe al conjunto de los factores biológicos, del comportamiento, conductuales y sociales, y no a uno de estos en particular.

Estos hallazgos están en concordancia con los de otros estudios longitudinales como el de Chao, *et al.* (2014), en el cual se encontró que la discapacidad física considerada desde la perspectiva de la reducción de la capacidad para realizar las actividades sociales habituales, así como el poco apoyo social y el incremento en el estrés percibido, contribuye al desarrollo de síntomas depresivos en los adultos mayores ^23^. Desde el punto de vista metodológico, se encontraron similitudes con el estudio citado en cuanto a la evaluación de la discapacidad mediante las limitaciones para las actividades básicas de la vida diaria, la presencia de síntomas depresivos con la escala CES-D en su versión de 10 ítems, y las diferencias en las variables consideradas como de confusión, toda vez que solo se incluyeron la edad, el sexo, el grado de escolaridad y el estado cognitivo.

La asociación entre discapacidad y síntomas depresivos también fue analizada por Chang, *et al.* (2009), quienes encontraron que el empeoramiento del nivel de discapacidad en mujeres de 65 años o más se asoció con un incremento de 2,2 veces más en la probabilidad de desarrollar síntomas depresivos en el corto plazo (OR=2,2; IC_95%_ 1,1-4,3), en este caso, un tiempo inferior a seis meses. En este estudio, las limitaciones para la realización de las actividades básicas de la vida diaria se utilizaron como indicador de la discapacidad y la presencia de síntomas depresivos se valoró con la *Geriatric Depression Scale* (GDS). Una diferencia metodológica entre el presente estudio y el de Chang, *et al.,* fue la exclusión de las personas con déficit cognitivo en esta última ^24^.

Por otra parte, Zeiss, *et al.* (1996), analizaron la asociación entre la discapacidad y los síntomas depresivos estratificando la muestra según el grado de discapacidad en personas que no la tenían (grupo 1), otras con discapacidad leve (grupo 2) y un tercer grupo (grupo 3) con discapacidad grave, y encontraron que 11, 13 y 21 %, respectivamente, desarrollaron dichos síntomas. Las comparaciones *post hoc* indicaron diferencias significativas entre los grupos 1 y 3 (p<0,001) y entre los grupos 2 y 3 (p<0,05) ^25^.

Prince, *et al.* (1998), por su parte, en su estudio prospectivo de base poblacional, documentaron que la discapacidad es uno de los predictores más relevantes para el desarrollo de depresión: la presencia de limitaciones para la realización de una a cuatro actividades básicas de la vida diaria se asoció con un incremento de 3,9 veces en el riesgo de depresión (IC_95%_ 1,79,4) y de 4,3 veces (IC_95%_ 1,5 -12,3) entre aquellos con limitaciones para la realización de cinco o más de dichas actividades en un modelo ajustado por edad, sexo, estado civil y redes de apoyo social ^26^.

Por otra parte, recientemente He, *et al.* (2019), registraron el porcentaje de quienes desarrollaron síntomas depresivos entre personas de mediana edad y adultos mayores de una población en China. En la encuesta de seguimiento de aquellos con puntuaciones iniciales de limitación para la realización de las actividades básicas de la vida diaria de 0,1 y ≥2, la incidencia de síntomas depresivos fue de 17,2 % (IC_95%_ 15,7-18,8 %), 22,3 % (IC_95%_ 18,1-26,6 %) y 34,8 % (IC_95%_ 27,5-42,1 %), respectivamente. Los sujetos con puntuaciones iniciales de 1 y ≥2 tuvieron un 38 % (OR=1,38; IC_95%_ 1,07-1,78) y 2,56 veces (OR=2,56; IC_95%_ 1,85-3,55) mayor riesgo de presentar síntomas depresivos. Después del ajuste por otras covariables sociodemográficas, de apoyo social y de estado de salud, la proporción de probabilidades disminuyó, pero la relación mantuvo significación estadística en el grupo con una puntuación inicial de ≥2 (OR=1,63; IC_95%_ 1,03-2,57) para la realización de las actividades básicas, a diferencia del grupo con una puntuación inicial de 1 (OR=0,99; IC_95%_ 0,73-1,35) ^27^.

Por otro lado, los resultados del presente estudio contrastan con los hallazgos del estudio longitudinal de Schieman, *et al.* (2007), en el cual la asociación entre discapacidad y síntomas depresivos variaba de acuerdo con el estrato socioeconómico, el sexo y la raza. En ese estudio, se observó que el incremento en el grado de limitaciones físicas se asociaba significativamente con la modificación hacia el incremento de la depresión entre adultos mayores de raza blanca con bajo nivel socioeconómico; por otra parte, entre aquellos con alto nivel socioeconómico, dicha asociación se mantuvo solamente en mujeres de raza negra y en hombres, independientemente de su raza ^28^.

No obstante, debe señalarse que existen diferencias metodológicas importantes con el presente estudio: la evaluación de los síntomas depresivos con una escala modificada de la *Hopkins Symptom Checklist for Depression* y la de discapacidad con un instrumento compuesto por elementos tomados de diversas escalas, incluida la de Katz. Además, en la selección de los participantes estos autores optaron por maximizar la diversidad social y económica, es decir, una población de estudio más heterogénea que la del presente estudio.

En un estudio de cohorte llevado a cabo en alemanes de 75 años o más, en el que se realizaron evaluaciones cada 1,5 años durante nueve años con base en el modelo de discapacidad de Verbrugge y Jette (1994), el cual considera que los eventos mórbidos agudos o crónicos que comprometen el estado físico de una persona se acompañan de cambios en la esfera psicológica que se traducen en síntomas depresivos, se encontró una asociación entre la modificación del nivel de discapacidad y dichos síntomas en el análisis bivariado (p<0,05), lo que no se mantuvo en el análisis multivariado ^29^. En este estudio, la discapacidad se midió con base en las limitaciones para la realización de las actividades básicas de la vida diaria y la depresión, con la *Geriatric Depression* Scale, y se consideraron como variables de confusión las sociodemográficas y otras relacionadas con la condición de salud que incluyeron 28 enfermedades.

En suma, tal y como lo describe Bruce, si bien es cierto que la conexión entre depresión y discapacidad podría ser intuitivamente obvia, los datos existentes sobre la naturaleza de esta relación sugieren que los vínculos entre los dos eventos son sorprendentemente complejos ^12^, lo que hace necesario mantener el incentivo para llevar a cabo investigaciones como la presente, con el propósito de contribuir a esclarecer la red causal e idear estrategias de prevención que mitiguen la carga que supone para los diferentes sistemas sanitarios y para la sociedad en general la atención de los adultos mayores con discapacidad y depresión.

Asimismo, es importante resaltar la importancia de este estudio en contextos como el mexicano, cuyo sistema de salud carece de directrices efectivas que garanticen la atención integral de una población, como la de los adultos mayores, doblemente vulnerable por la presencia de la discapacidad y la depresión, y en la cual frecuentemente se subestima la presencia de depresión e, incluso, se la llega a considerar como normal.

Por otro lado, según lo reportado por la Organización Mundial de la Salud (OMS), el 80 % de las personas con discapacidad viven en países de ingresos bajos o medios, como los de Latinoamérica ^5^. Allí, a diferencia de los países con altos ingresos, son escasos los datos sobre la prevalencia e incidencia de la depresión en las etapas tardías de la vida y sobre los factores de riesgo potencialmente modificables relacionados con este evento. Esta situación podría deberse a distintas razones como la diversidad cultural, la notificación o conceptualización de los síntomas depresivos, los factores psicométricos, los diferentes perfiles de factores de riesgo o las diferencias en las características socioeconómicas ^30^.

Por último, cabe mencionar como fortalezas del estudio, su enfoque longitudinal, el hecho de contar con una muestra representativa a nivel nacional, el control en el análisis multivariado de múltiples factores de confusión y la evaluación de la relación entre la discapacidad y los síntomas depresivos clínicamente significativos y diferentes indicadores de limitación de actividades, lo que permitió establecer que el efecto se mantiene independientemente del indicador de discapacidad que se analice. Uno de los factores que mejor podría explicar los resultados disímiles de los diversos estudios sobre la relación entre discapacidad y síntomas depresivos, es el uso de diferentes métodos para medir dichas variables.

Entre las limitaciones, se encuentran las pérdidas en el seguimiento, la evaluación de las principales variables de interés con base en el propio reporte de los participantes, la presencia de potenciales variables de confusión no medidas, y el hecho de que, dadas las características de la escala CES-D, solo se tuvieron en cuenta aquellas personas con síntomas depresivos recientes, específicamente en la semana inmediatamente anterior, lo que podría haber llevado a pasar por alto casos incidentes por fuera de este periodo.

En cuanto a las pérdidas en el seguimiento, debe tenerse en cuenta que este estudio se circunscribe a los datos disponibles en la encuesta ENASEM. Entre las rondas de estudio se registraron 239 muertes y solo una de las pérdidas en el seguimiento se registró en ese grupo. Ciertamente, es complejo conocer todas las causas que motivaron dichas pérdidas, sin embargo, el análisis incluyó varias variables independientes reconocidas en la literatura como causas comunes de muchos resultados de salud que podrían estar relacionadas con las pérdidas. Por otro lado, la proporción de personas expuestas a la discapacidad fue mayor entre los sujetos que no pudieron ser seguidos, por lo que habría que considerar que tanto la incidencia de síntomas depresivos clínicamente significativos como la fuerza de asociación entre la discapacidad y este evento, podrían haberse subestimado.

Es preciso reconocer el aumento del riesgo de síntomas depresivos clínicamente significativos entre los adultos mayores con discapacidad. Dada su alta incidencia y el incremento esperado de este grupo de población, es necesario implementar y adaptar las políticas preventivas de los diversos sistemas de salud, máxime en contextos como el latinoamericano debido a los altos costos financieros derivados de la atención de adultos mayores con síntomas depresivos clínicamente significativos.
